# Self‐Assembled Monolayer Anode Enables 7% Efficiency in Y6‐Based Quasi‐Homojunction Solar Cells With 4% Donor Contents

**DOI:** 10.1002/smsc.202500552

**Published:** 2026-03-19

**Authors:** Man Hing Suen, Zhuoqiong Zhang, Yidan An, Pengyu Du, Yunfan Wang, Yu Tang, Chujun Zhang, Shibo Wang, Tanghao Liu, Shanchao Ouyang, Guilong Cai, Guanghao Lu, Hin‐Lap Yip, Sai Wing Tsang, Shu Kong So

**Affiliations:** ^1^ Department of Physics and Institute of Advanced Materials Hong Kong Baptist University Hong Kong SAR China; ^2^ Department of Materials Science and Engineering City University of Hong Kong Hong Kong SAR China; ^3^ School of Chemistry Xi’an Jiaotong University Xi’an China; ^4^ Hunan Key Laboratory for Super‐microstructure and Ultrafast Process School of Physics Central South University Changsha China; ^5^ Beijing Key Laboratory of Ionic Liquids Clean Process Institute of Process Engineering Chinese Academy of Sciences Beijing China; ^6^ School of Physical Sciences Great Bay University Dongguan Guangdong China

**Keywords:** homojunction cells, organic solar cells, quasi‐homojunction, self‐assembled monolayer, vertical phase separation

## Abstract

Quasi‐homojunction (QHJ) organic solar cells (OSCs) offer a promising alternative architecture that combines the advantages of bulk heterojunction (BHJ) and homojunction (HJ) designs. By blending a minimal fraction of donor material (a few wt%) into a nonfullerene acceptor matrix, QHJ devices can be designed to achieve efficient charge separation and transport while avoiding the morphological complexity and instability of BHJs. This study demonstrates that Y6‐based QHJ OSCs, incorporating only 4 wt% donor content, achieve a power conversion efficiency of 7.1%. This performance enhancement is enabled by replacing the PEDOT:PSS anode with a novel self‐assembled monolayer anode, which induces vertical phase separation, positioning the donor polymer at the anode interface to enhance charge extraction. The optimized vertical morphology not only facilitates efficient charge transport but also ensures excellent stability, maintaining consistent performance across active layer thicknesses of 55–180nm. This highlights the potential of QHJ architecture to combine the simplicity of HJ with the performance advantages of BHJ.

## Graphical abstract

Quasi‐homojunction (QHJ) organic solar cells combine the simplicity of homojunctions with the performance benefits of bulk heterojunctions. Using only 4 wt% donor material and a novel self‐assembled monolayer anode, Y6‐based QHJ devices achieve 7.1% efficiency. The optimized vertical phase separation enhances charge extraction and stability, offering a robust design across varying active layer thicknesses.

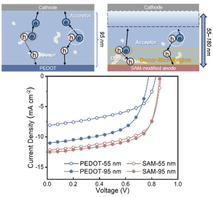

## 1 Introduction

**FIGURE 1:**
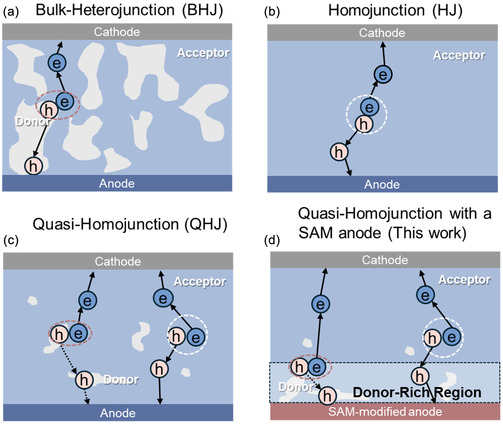
Illustration of four distinct active layer morphologies: (a) BHJ, (b) HJ, (c) QHJ, (d) QHJ with a SAM anode (this work). (a) and (c) Adapted from reference with permission [1], 2025, John Wiley & Sons. Hole transport in the QHJ is governed by two primary mechanisms: tunneling between donor domains (dotted line) and transport through the acceptor phase (solid line). In (a) and (b), the predominant exciton dissociation pathways occur at a donor/acceptor interface (dotted ellipse) and in the bulk (dotted circle), respectively. In (c) and (d), both dissociation pathways are viable.

Organic solar cells (OSCs) based on bulk heterojunction (BHJ) architecture (Figure 1a) have attracted considerable interest in recent years due to the development of nonfullerene acceptors (NFAs). Nowadays, power conversion efficiency (PCE) of BHJ devices can exceed 20% [2, 3, 4, 5]. In such kind of BHJ cells, a donor polymer is typically blended with an electron acceptor—often serves as the light absorber—in approximately equal proportions to establish a morphology for effective charge separation and transport. This morphology must balance two competing requirements: maximizing donor–acceptor interfacial area to enhance exciton dissociation and separation, while maintaining phase separation large enough to ensure continuous pathways for bipolar charge transport [6, 7]. These conflicting demands on the morphology create an intrinsic dilemma and often result in built‐in instability of BHJ cells—an issue that still requires substantial efforts to resolve [8, 9].

In contrast to BHJ cells, homojunction (HJ) OSCs (Figure 1b) can be designed using a single material component for light absorption, charge generation, and charge transport [10, 11, 12, 13, 14]. This design concept is appealing, and the complex mixed film morphology of the active layer is no longer an issue [11, 12, 13]. However, HJ cells are not considered to be practical at all as they have very low efficiencies due to poor charge generation and severe charge recombination. With the emergence of Y‐acceptors, HJ cells acquire renewed attention due to the possibility of enjoying much higher free charge generation in these NFAs [10, 11, 13, 15]. Notably, recent HJ studies have reported PCEs of 4.4% in Y6 [12] and 4.2% in L8BO‐based OSCs [16]. Despite these promising results, NFA‐based HJ OSCs still suffer from substantial bulk and interface recombination, and their PCEs remain far below the 20% benchmark achieved by BHJ cells [11, 12, 13].

In parallel with HJ cells, quasi‐homojunction (QHJ) cells are being explored, having the potential of delivering better PCEs than HJ cells while maintaining simplified morphology. In a QHJ cell (Figure 1c), a small amount of donor material (typically a few wt%) is introduced into the host acceptor, which still dominates the optical absorption [1]. Despite present in a small fraction, the donor can facilitate exciton dissociation and charge extraction, leading to enhanced PCEs. An early demonstration of QHJ cells was reported by Tang in 2011 [17]. Subsequent studies adopted the terminologies of ‘dilute’ or ‘low‐donor’ BHJ cells [18, 19, 20, 21, 22, 23]. In 2022, Zhan's group formally introduced the terminology of QHJ cells by using Y6 as the primary acceptor material, blended with ≈5% donor polymer [1]. In Y6‐based HJ cells, a very low PCE of 0.052% was obtained, but with a much‐enhanced PCE of 4.18% was achieved in QHJ cells with 5 wt% PM6 donor content. In Zhan's work, highly conductive PEDOT:PSS was adopted as the anode layer, which is in direct contact with the active layer. This anode layer works well for conventional BHJ cells. But in a HJ or QHJ cell, it is problematic as the absence of sufficient donor materials leads to significant minority carrier (electron) capture at the anode/active layer interface [16]. Further studies are definitely needed to improve the anode and unlock the full potential of QHJ cells.

In this study, we replaced the PEDOT:PSS anode in a QHJ cell with a dual layer film consisting of a self‐assembled monolayer (SAM), namely, Cbz‐2Ph on a nickel oxide (NiO_x_) anode. For clarity, we will refer to these materials as PEDOT and SAM, respectively, throughout the manuscript. We achieved, in a QHJ cell consisting of Y6 and 4% PM6 donor, a high PCE of 7.1%, with an open‐circuit voltage (*V*_OC_) of 0.862 V and a fill factor (FF) of 0.65 with the SAM‐based anode. Meanwhile, the PEDOT‐based QHJ structure exhibited a lower PCE of 4.7%, with a *V*_OC_ of 0.772 V and an FF of 0.59. Similar observations were also made in other donor additives in Y6‐based QHJ cells. Comprehensive analysis, including surface energy analysis, Kelvin probe force microscopy (KPFM), ultraviolet photoelectron spectroscopy (UPS), photoluminescence (PL), and film‐depth‐dependent light absorption spectroscopy (FLAS), revealed key insights into the interaction between the QHJ active layer and the SAM anode in QHJ cells. Unlike PEDOT, the SAM anode enhanced vertical phase separation within the QHJ structure (Figure 1d), positioning the donor polymer closer to the hole transport layer (HTL) interface. This optimized configuration contributes significantly to improved device performance. Furthermore, this SAM‐based QHJ cell maintained a stable PCE across a wide active layer thickness range (55–180 nm), demonstrating remarkable thickness tolerance, which greatly relaxes the stringent processing constraints for commercial production in OSCs. This work establishes that the buried interface is a critical, yet under‐explored, avenue for maximizing the potential of QHJ OSCs. The SAM anode not only enhances OSC performance significantly but also holds strong potential for applications in flexible, semitransparent electronics, and manufacturable OSCs, advancing their suitability for real‐world deployment [24].

## 2 Results and Discussion

### 2.1 Device Performance of HJ and QHJ OSCs

**FIGURE 2:**
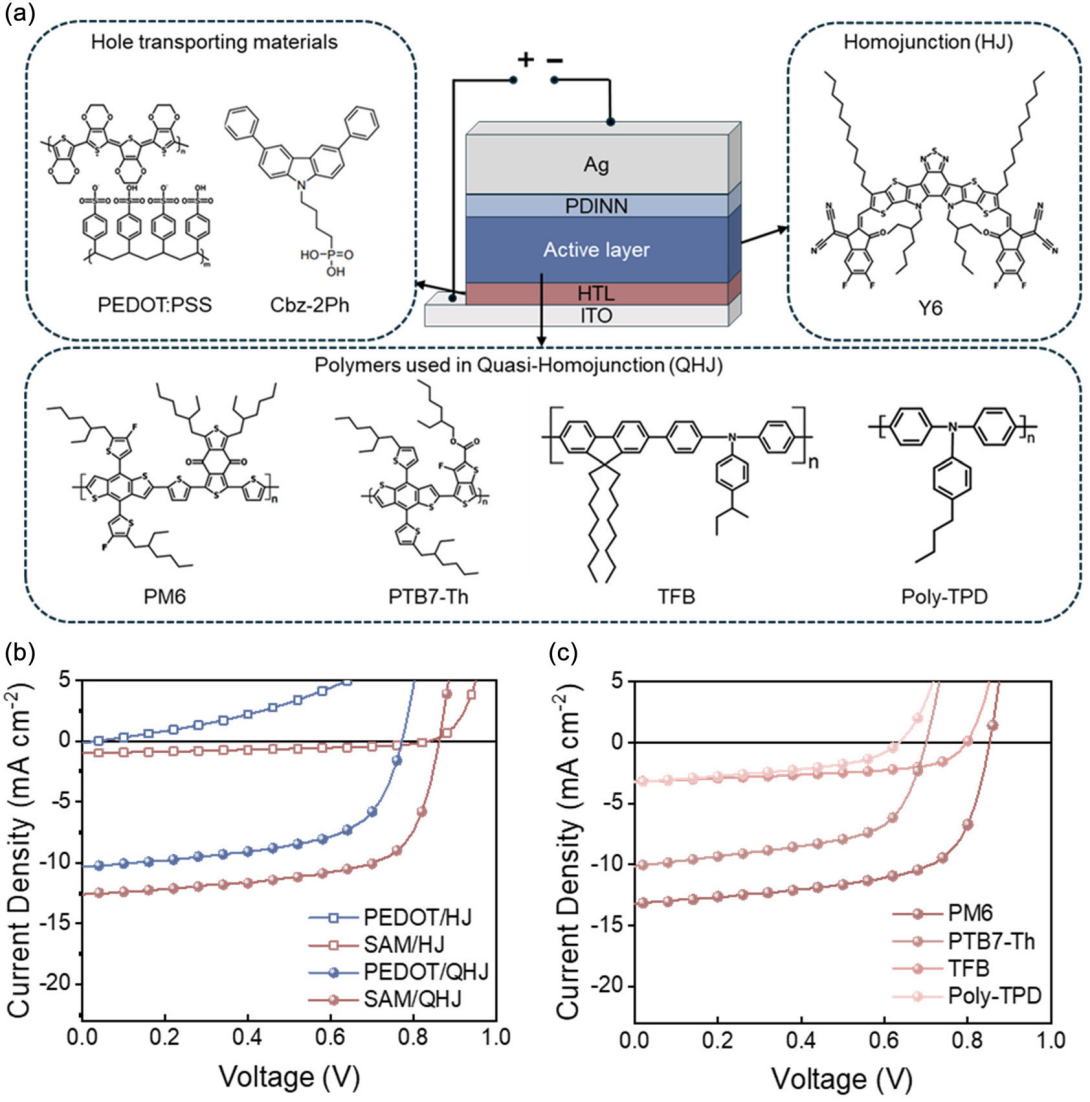
(a) Schematic of OSC architecture, highlighting two HTL materials: PEDOT:PSS and Cbz‐2Ph, with their respective chemical structures. The active layer comprises Y6 blended with four polymer additives: PM6, PTB7‐Th, TFB, and Poly‐TPD, (b) *J–* *V* characteristics of HJ and QHJ PM6:Y6 OSCs using PEDOT and SAM as anodes, (c) *J–* *V* characteristics of QHJ devices with SAM‐based anodes incorporating various polymers.

Y6‐based HJ or QHJ OSCs were fabricated on either a PEDOT or a SAM‐coated indium‐tin‐oxide (ITO) anode. The SAM anode layer was prepared by two steps. First, a NiO_x_ film was deposited on ITO *via* spin‐coating and annealing. Second, the dried NiO_x_ film was spin‐coated with a novel SAM, namely, Cbz‐2Ph. The resulting interlayer facilitates strong van der Waals contact with the donor polymer and leverages a large molecular dipole moment to tune the work function (WF), optimizing both hole extraction and maximize photovoltage [25]. The overall device was ITO/HTL/active layer/PDINN/Ag. HJ cells were prepared by spin‐coating Y6 on the anode followed by the cathode layers of PDINN/Ag deposition. QHJ cells were prepared by blending Y6 with a small dosage of donor polymer, which included choice of PM6, PTB7‐Th, TFB, or Poly‐TPD. These polymers, widely recognized for their high hole mobility and broad applicability in OSCs and organic light‐emitting diodes, are expected to minimize the charge recombination within the active layer [26, 27, 28]. The detailed structure of the OSC, along with the chemical structures of the HTLs, Y6, and polymer additives, is shown in Figure 2a. Fabrication details are provided in the Supporting Information. The current density–voltage (*J*–*V*) characteristics and corresponding photovoltaic parameters of both HJ and QHJ OSCs with different anodes are shown in Figure 2b and Supporting Information Table S1.

In general, the Y6 HJ on PEDOT anode devices show extremely inferior performance, with *V*_OC_ and short‐circuit current density (*J*_SC_) values approaching zero. The poor performance can be attributed to significant bimolecular and trap‐assisted recombination in the active layer, as suggested by previous studies [10, 11, 15]. When the anode was switched to SAM, the HJ cells saw a significant increase in *V*_OC_ from 0.073 to 0.840 V. *J*_SC_ and FF also increased from 0.2 to 1.0 mA cm^−2^ and 0.27 to 0.40 (detailed in Figure 2b and Supporting Information Table S1), respectively. The increase in *J*_SC_ is primarily influenced by light absorption and charge collection efficiency. Given that PEDOT and SAM anodes exhibit comparable transmittance properties (Supporting Information Figure S1), the enhanced *J*_SC_ in SAM‐based HJ devices is mainly from the improved charge collection efficiency [25]. However, despite these advancements, the *J*_SC_ values in both PEDOT‐ and SAM‐based HJ devices remain relatively low. In contrast to HJ cells, the Y6‐based QHJ cells enjoy much higher performance. Our QHJ cells were purposely chosen to contain 4% of a selected donor polymer within Y6 active layer. This donor content is below the typical percolation threshold (10–30 wt%) when the polymer blended into a host [29]. In this way, the added PM6 plays a negligible role in hole transport. Its primary function is to facilitate exciton dissociation and suppress charge recombination. For QHJ cells on the PEDOT anode, significant improvements were observed in *J*_SC_, *V*_OC_, and FF, with values of 10.3 mA cm^−2^, 0.772 V, and 0.59, respectively, as detailed in Figure 2b and Supporting Information Table S1. These improvements indicate that the 4 wt% donor additives can effectively reduce the charge recombination in the active layer. In comparison, SAM‐based QHJ devices demonstrated even greater improvements, with a *J*_SC_ of 12.6 mA cm^−2^, a *V*_OC_ of 0.862 V, and an FF of 0.65 (the integrated photocurrent from the external quantum efficiency data is consistent with the *J*_SC_ value, as shown in Supporting Information Figure S2). This improvement indicates that an appropriate donor additive can effectively reduce the charge recombination.

**FIGURE 3:**
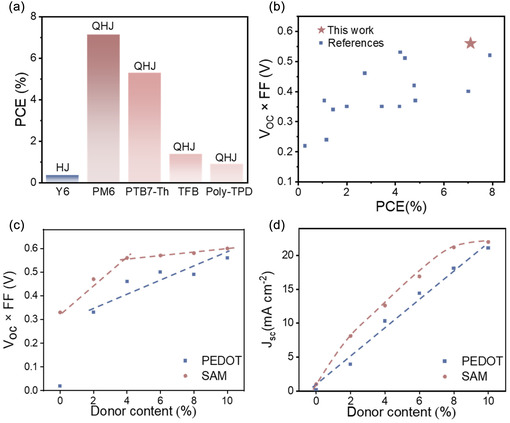
(a) PCE distribution of SAM‐based HJ and QHJ OSCs with different donors. (b) Comparison of the PCE and *V*_OC _× FF values of HJ and QHJ OSCs reported in the literature. Variation of (c) *V*_OC _× FF and (d) *J*_SC_ for PM6:Y6‐based OSCs with varying donor contents.

Besides PM6, the universality was verified by blending Y6 with other p‐type polymers such as PTB7‐Th, TFB, and Poly‐TPD. The results were summarized in Figure 2c and Supporting Information Table S2. Compared to the HJ devices, the incorporation of polymer additives notably enhanced photovoltaic performance, particularly the *J*_SC_ and FF, underscoring the effectiveness and versatility of the QHJ strategy with SAM anode. The variation in *V*_OC_ observed among the devices is attributed to differences in energy level (Supporting Information Figure S3). Among the various additives, SAM‐based QHJ cells incorporating PM6 achieved the highest PCE of 7.1% (Figure 3a). Therefore, the subsequent analysis primarily focuses on the PM6 incorporated SAM‐based QHJ cells.

To evaluate the performance of our QHJ device, we compared its photovoltaic parameters with those of other HJ and QHJ cells reported in the literature, focusing on studies with donor contents below 5%. To benchmark our QHJ device performance, we made a plot of *V*_OC_ × FF against PCE. The product *V*_OC_ × FF can be understood as the maximum power output per unit of photogenerated current, serving as a combined measure of contact quality (*via* *V*_OC_) and charge extraction (*via* FF). As summarized in Figure 3b (with additional details in Supporting Information Table S3), our SAM‐based QHJ device demonstrated notably high PCE (7.1%) and *V*_OC_ × FF (∼0.56 V), surpassing the performance of most recently reported HJ and QHJ cells. The high *V*_OC _× FF observed in our devices mainly reflects efficient charge extraction and suppressed recombination at the active layer and the anode/active layer interface [30, 31, 32].

To investigate the critical role of the anode in mitigating charge recombination in QHJ devices, we conducted a detailed analysis by systematically varying the donor contents in the Y6‐based active layer from 0% to 10%, using PEDOT and SAM as anodes. Figure 3c,d show the trends in *V*_OC_ × FF and *J*_SC_ as a function of donor content in OSCs, with detailed *J–* *V* characteristics, photovoltaic parameters, and optical transmittance data provided in Supporting Information Figures S4–S7, and Table S4. At 0 wt% donor content, SAM‐based devices exhibited a significantly higher *V*_OC_ × FF value of 0.336 V compared to only 0.020 V for PEDOT‐based devices. This stark difference highlights the poor performance of PEDOT as an anode in the HJ system. For PEDOT‐based devices, *V*_OC_ × FF starts to increase sharply between 0% and 2% donor content and subsequently displays a gradual linear rise from 2% to 10%. This linear trend closely corresponds to the linear growth in *J*_SC_, as shown in Figure 3d. The performance improvement is consistent with an expanded donor–acceptor interfacial area as the donor content increases [33]. In contrast, SAM‐based devices demonstrate a more pronounced rise in *V*_OC_ × FF within the 0%–4% donor content range, followed by a slower, more gradual rise at higher donor levels (Figure 3c). The transition point at 4% donor content marks a noticeable change in the *V*_OC_ × FF growth trend. This shift may reflect the point at which donor content becomes sufficient to promote enhanced charge generation and extraction efficiency, leading to a reduced sensitivity to further increases in donor concentration. Beyond 4% donor content, *V*_OC_ × FF tends to saturate where further donor addition contributes less to enhancing the product. The steeper initial increase in *J*_SC_ observed in SAM‐based devices compared to PEDOT‐based devices (Figure 3d) further highlights the superior charge transport properties of SAM‐based systems, particularly at lower donor contents.

This distinction underscores the role of the SAM anode in facilitating efficient charge extraction and mitigating recombination in OSCs. Notably, SAM‐based devices exhibit a transition point in *V*_OC_ × FF at a donor content as low as 4 wt%. This transition point occurs earlier than typically reported in prior studies (≈10% donor content, as demonstrated in Supporting Information Figure S8) [1, 34]. Such differences may be attributed to the ability of the SAM anode to promote efficient charge extraction and transport even at relatively low donor concentrations.

### 2.2 How a SAM Anode Promotes Polymer Phase Separation in the QHJ Active Layer

**FIGURE 4:**
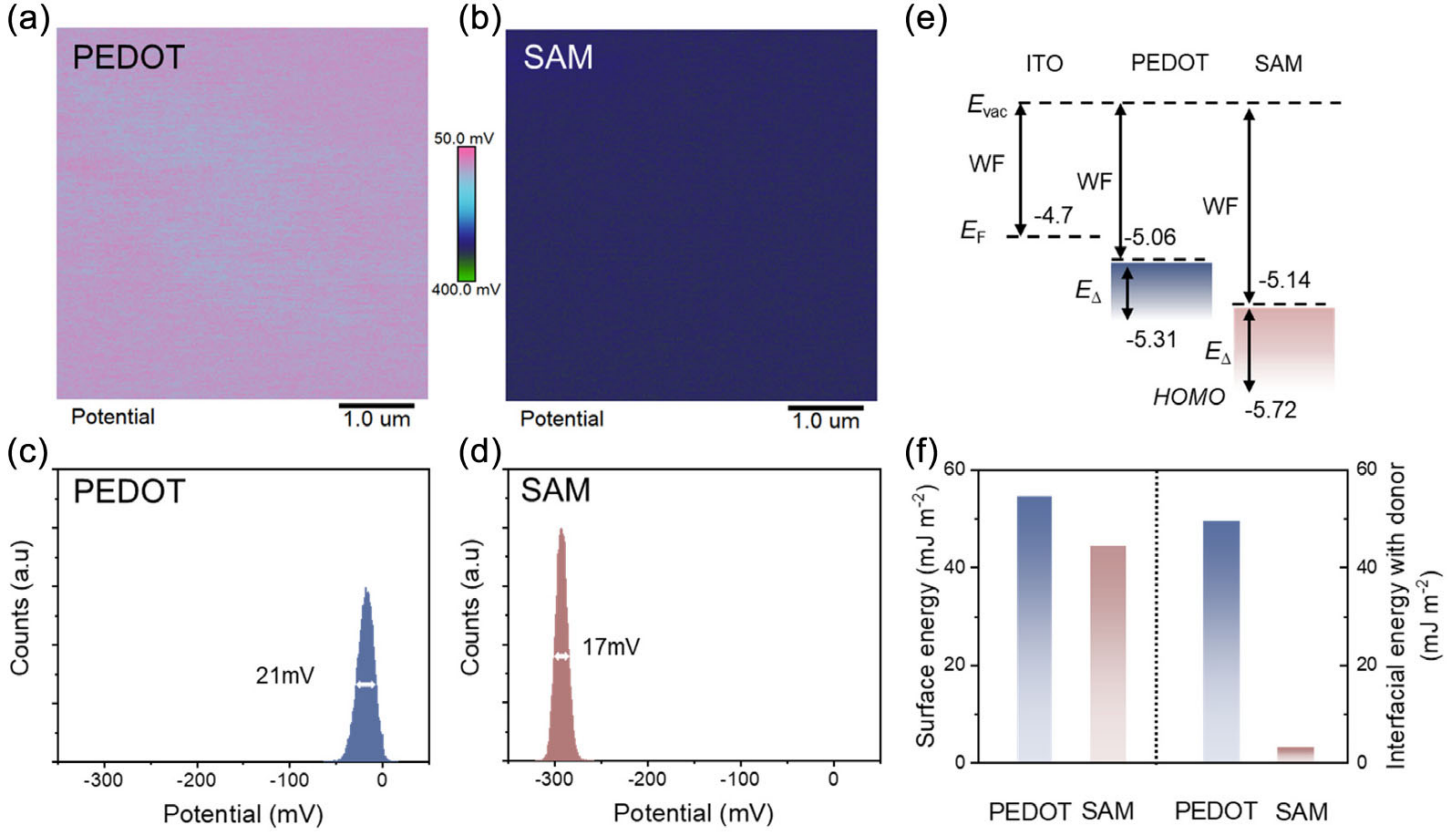
(a) KPFM images of PEDOT‐ and (b) SAM‐based films. Corresponding potential distribution plots for (c) PEDOT‐ and (d) SAM‐based films, respectively. (e) The energy‐level diagram of ITO, PEDOT, and SAM derived from the UPS measurement, where *E*_vac_, *E*_F_, and *E*_Δ_ represent vacuum level, Fermi energy level, and energy gap between *E*_F_ and highest occupied molecular orbital (HOMO) of PEDOT or SAM. (f) Calculated surface energies of PEDOT and SAM anodes, along with their interfacial energies with the PM6 material.

To explore the interfacial effects of PEDOT and SAM anodes, their properties were analyzed using KPFM, UPS, and surface tension measurements. The surface potential maps of PEDOT and SAM films, captured by KPFM, are shown in Figure 4a,b, with the corresponding contact potential difference (CPD) distributions across the scan areas presented in Figure 4c,d. The SAM film exhibited a narrower full width at half maximum (FWHM) of 17 mV compared to 21 mV for the PEDOT film, suggesting a more uniform surface morphology, which is advantageous for enhancing the optoelectronic performance of OSCs [35]. In addition, the SAM film displayed a significantly lower CPD of **≈**−290 mV compared to −30 mV for the PEDOT film (Figure 4a,b), suggesting a deeper WF for the SAM anode. This finding is further supported by UPS measurements, which confirmed a deeper WF for the SAM anode (−5.14 eV) compared to the PEDOT anode (−5.06 eV), as shown in Figure 4e and Supporting Information Figure S9. The deeper WF of the SAM anode enables more favorable energy level alignment at the interface, which could contribute to improved charge extraction in the OSCs_._

We also analyzed the surface energy of HTLs and their interfacial tension with the donor polymer to understand their influence on the morphology of the active layer [36, 37, 38, 39]. Contact angle tests were performed using deionized water and diiodomethane droplets on PEDOT and SAM surfaces, with the results summarized in Supporting Information Table S5 and Figure S10. Surface energies were calculated using the Wu model (harmonic mean) based on the contact angle data [39, 40], and are presented in Figure 4f (details in Supporting Information). The SAM surface exhibited a lower surface energy of 44.3 mJ m^−2^ compared to 54.6 mJ m^−2^ for PEDOT surface. This reduction in surface energy is expected to lower interfacial resistance between the ITO electrode and the active layer, which may contribute to the improved FF observed in SAM‐based devices [41, 42]. We further examined the interfacial energy between the HTLs and donor polymer PM6, to assess how the surface tension of HTLs affects polymer phase stratification within the active layer (detailed in Supporting Information) [39]. Interfacial energy is minimized when the dispersion and polar components of the two materials are closely aligned, promoting better interaction [43]. As shown in Figure 4f, the interfacial tension between PM6 and SAM anodes was significantly lower (3.1 mJ m^−2^) compared to that between PM6 and PEDOT anodes (49.4 mJ m^−2^). This lower interfacial tension for the SAM anode indicates a stronger interaction and enhanced compatibility between the SAM and PM6, leading to improved adhesion and a more stable interface [39, 43]. Such compatibility may influence the morphology of the active layer, potentially inducing the formation of vertical phase separation, where PM6 preferentially aligns at the anode interface [38, 44]. This improved morphology can facilitate more efficient charge transport pathways and suppress charge recombination, contributing to the improved device performance observed in SAM‐based architectures.

**FIGURE 5:**
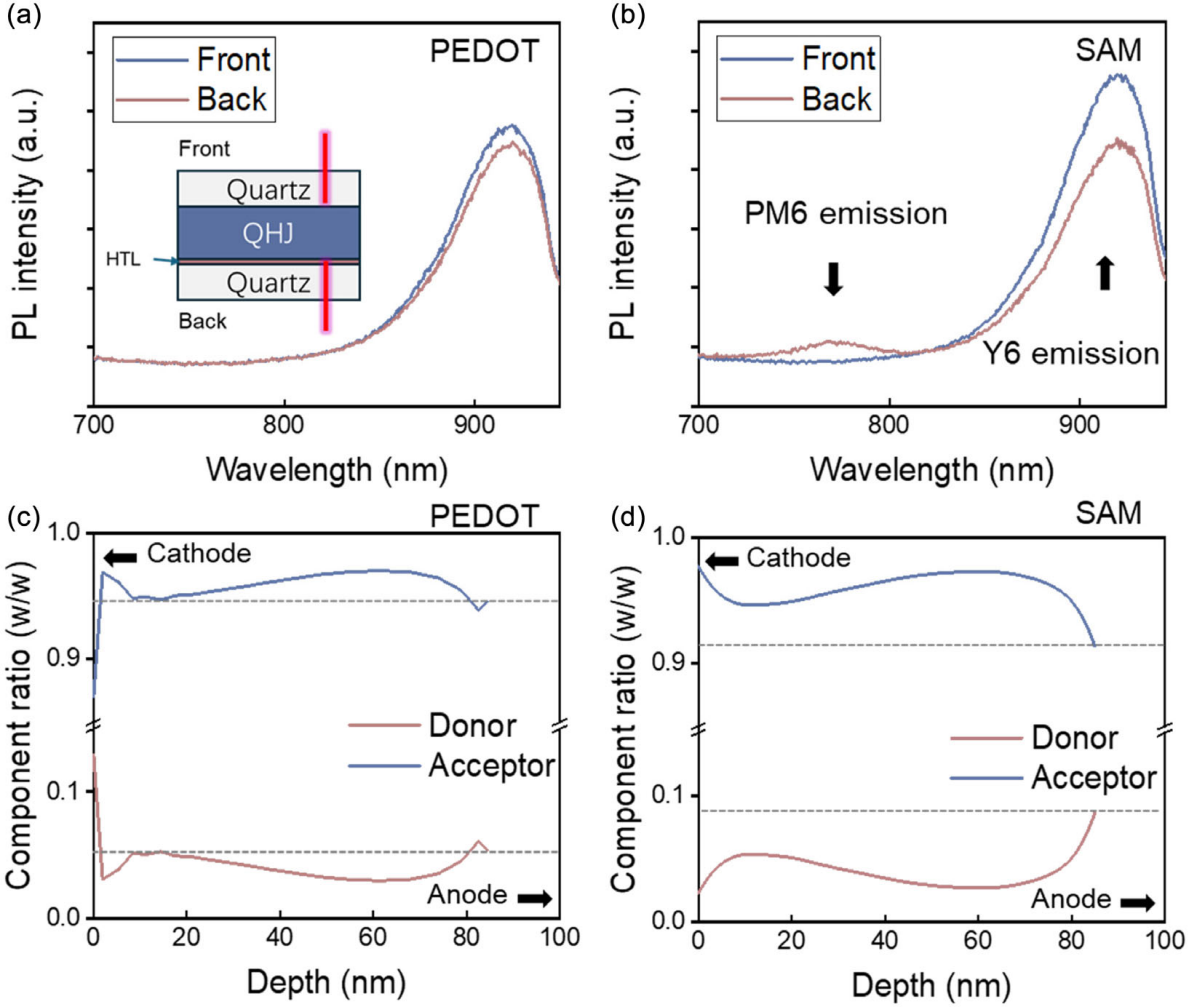
PL spectra measured from the front and back sides of (a) PEDOT‐ and (b) SAM‐based films. The inset illustrates a schematic diagram of the experimental setup for front and back PL measurements. FLAS analysis shows the calculated compositional variation profiles of blend films with (c) PEDOT and (d) SAM as anodes.

In order to investigate the vertical distribution of the QHJ active layer on different anodes, PL spectra were measured from both the front (active layer side) and back (anode side) of QHJ films deposited on PEDOT and SAM anodes, as shown in Figure 5a,b. A front quartz substrate was used in the sample configuration to maintain a consistent distance between the active layer surface and the measurement apparatus in both configurations, ensuring reliable comparisons. Both PEDOT‐ and SAM‐based QHJ samples exhibited strong PL emission peaks at 920 nm, consistent with the Y6 reference peak (Supporting Information Figure S11) [45]. For PEDOT‐based samples (Figure 5a), the PL spectra measured from the front and back sides were nearly identical, indicating a uniform composition throughout the film. In contrast, SAM‐based samples (Figure 5b) exhibited an additional prominent PL peak between 700 and 800 nm when measured from the back side, corresponding to the PM6 emission peak (Supporting Information Figure S11) [45]. This result suggests a higher concentration of PM6 at the bottom of the SAM‐based QHJ layer compared to its top surface, highlighting a distinct vertical distribution in SAM‐based samples that differs from the uniformity observed in PEDOT‐based samples.

Building on the observed differences in vertical distribution, FLAS was utilized to gain deeper insight into the composition of the active layer on PEDOT and SAM anodes [46, 47, 48, 49]. This technique provides a detailed profile of donor and acceptor material distribution across the film thickness. The detailed FLAS for QHJ film with PEDOT and SAM as anodes are shown in Supporting Information Figure S12. Using the absorption spectrum overlapping method, the relative distributions of PM6 and Y6 were quantified at different depths within the active layer (Figure 5c,d) [50]. Here, a depth of 0 nm represents the interface near the cathode, while 85 nm corresponds to the interface adjacent to the anode (∼the total QHJ thickness). At the 85 nm depth, the SAM‐based sample exhibited a higher PM6 concentration of 8.7% and a lower Y6 concentration of 91.3%, compared to the PEDOT‐based sample, which had a PM6 concentration of 5.4% and a Y6 concentration of 94.6% at the same depth. This enrichment of PM6 near the anode in the SAM‐based sample is consistent with the surface tension analysis and PL spectra [44]. Building on these findings, a schematic model is proposed (Figure 1d). Unlike the configuration shown in Figure 1c, the SAM‐based anode leverages its unique interfacial properties to localize the PM6 donor material near the anode. This vertical phase separation likely contributes to improved device performance by promoting effective charge separation and minimizing recombination losses [51].

**FIGURE 6:**
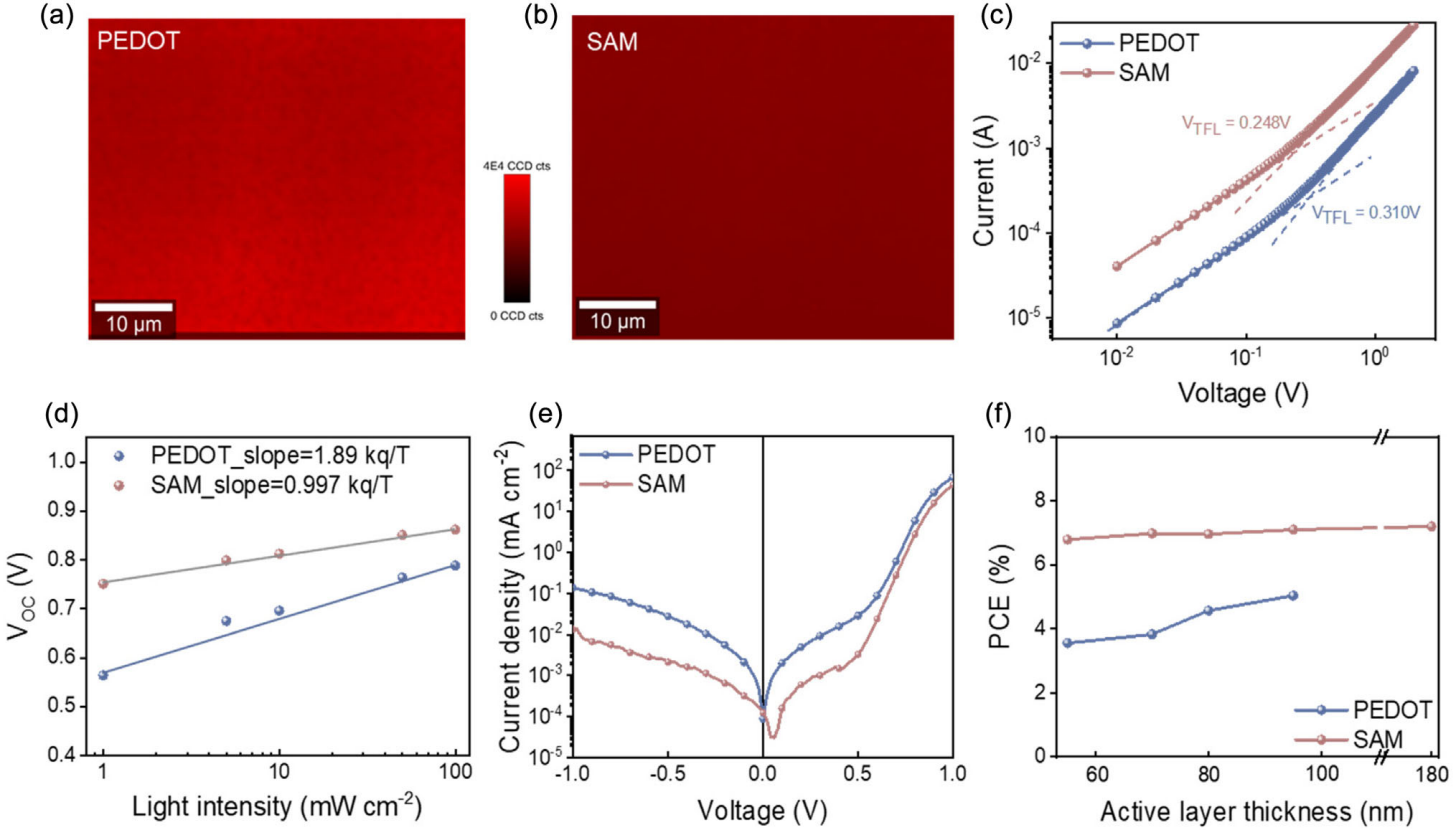
PL mapping of QHJ films on (a) PEDOT‐ and (b) SAM‐based anodes, respectively. (c) SCLC measurements of hole‐only devices with PEDOT and SAM as anodes. (d) Light intensity dependence of *V*_OC_ for OSCs with PEDOT‐ and SAM‐based anodes. (e) Dark current of QHJ OSC with PEDOT and SAM as anodes. (f) PCE as a function of active layer thickness for QHJ OSC with PEDOT‐ and SAM‐based anodes.

Furthermore, we performed confocal PL mapping to understand the correlation between morphology and carrier dynamics in QHJ films deposited on PEDOT and SAM anodes (Figure 6a,b). The SAM‐based QHJ sample exhibited a reduced and more homogenous PL intensity distribution compared to the PEDOT‐based film. This observation suggests improved film morphology and enhanced ability to extract free carriers at the interface in the SAM‐based system [52]. We further evaluated hole mobility in both systems using the space‐charge‐limited current (SCLC) method with hole‐only devices. As shown in Figure 6c, the SAM‐based devices demonstrated a higher hole mobility (1.14 × 10^−4^ cm^2^ s^−1^ V^−1^) compared to the PEDOT‐based devices (8.37 × 10^−5^ cm^2^ s^−1^ V^−1^). This enhancement in mobility indicates more efficient hole transport in SAM‐based devices and the overall photovoltaic performance. Additionally, we estimated the total trap density (*N*_t_) by analyzing the trap‐filling limit voltage (*V*_TFL_), which marks the transition between the ohmic and trap‐filled limited regions (detailed calculation in Supporting Information). The SAM‐based devices exhibited a lower *V*_TFL_ of 0.248 V and a reduced *N*_t_ of 9.12 × 10^15 ^ cm^−3^, compared to the PEDOT‐based devices, which had a *V*_TFL_ of 0.310 V and a *N*_t_ of 1.14 × 10^16^ cm^−3^ (Supporting Information Table S6). These results indicate fewer charge‐trapping defects in SAM‐based devices, which likely reduce recombination losses and improved hole extraction. The enhanced charge extraction capabilities were further supported by transient photocurrent measurements (Supporting Information Figure S13). The SAM‐based QHJ devices demonstrated a faster charge extraction time of 0.202 μs compared to 0.357 μs for the PEDOT‐based devices, indicating more efficient charge transport and extraction [39, 44]. These advantages can be attributed to the optimized interfacial properties and improved film morphology, reinforcing the role of SAM anode in enhancing device performance [53].

### 2.3 Charge Recombination in a QHJ Device With Vertical Phase Separation

To investigate recombination behaviors in devices using SAM and PEDOT as anodes, we conducted light‐intensity‐dependent measurements of both *V*_OC_ and *J*_SC_ (Supporting Information Table S7). The relationship between *V*_OC_ and light intensity follows a logarithmic trend, where the slope, expressed as *n*kT/*q*, is derived from the fitting line. Here, *n* represents the slope, k is the Boltzmann constant, *T* is the absolute temperature, and *q* is the elementary charge [54]. As shown in Figure 6d, SAM‐based devices exhibit a shallow slope of 0.997 kT/*q*, significantly lower than the 1.89 kT/*q* observed for PEDOT‐based devices. This indicates that the SAM anode effectively mitigates trap‐assisted Shockley–Read–Hall recombination, in agreement with the SCLC results [55, 56]. We also analyzed the dependence of *J*_SC_ on light intensity, which follows a power–law relationship, *J*_SC_ ∝ I^*α*^, where an *α* value close to 1 indicates minimal bimolecular recombination losses [57]. As shown in Supporting Information Figure S14, the *α* value for SAM‐based devices is 0.897, slightly higher than the 0.889 measured for the PEDOT‐based devices. This result indicates reduced bimolecular recombination in SAM‐based devices, correlating with enhanced FF and overall device performance. These findings are further supported by dark *J* *–* *V* measurements (Figure 6e), which show a reduced leakage current density for SAM‐based devices. Together, these results highlight the effectiveness of SAM in optimizing charge recombination processes, ultimately contributing to improved device efficiency.

### 2.4 Thickness Independence Enabled by Vertical Phase Separation in SAM‐Based QHJ Devices

The large‐scale manufacturing of OSCs will require tolerance to variations in active layer thickness to ensure reliable performance [58]. To evaluate this, we investigated the performance of SAM‐based devices under varying active layer thicknesses, ranging from 55 to 180 nm. Reducing the thickness to 55 nm diminishes the light absorption volume and exciton generation, making efficient charge extraction by the anode critical for maintaining performance. As shown in Figure 6f (with details in Supporting Information Figures S15–S17, and Table S8), PEDOT‐based devices exhibited a significant decline in performance at a 55 nm active layer, with a PCE of 3.6%, *J*_SC_ of 8.1 mA cm^−2^, *V*_OC_ of 0.832 V, and FF of 0.53. In contrast, SAM‐based devices maintained higher efficiency under the same conditions, achieving a PCE of 6.8%, *J*_SC_ of 12.2 mA cm^−2^, *V*_OC_ of 0.871 V, and FF of 0.64. The PCE can be maintained even for the thick film of ∼180 nm (Supporting Information Figure S17). The superior performance of SAM‐based devices at reduced thickness is attributed to their optimized vertical phase separation, which retains a PM6‐rich region near the anode. This configuration enhances charge extraction and minimizes recombination losses, even with fewer exciton generated. In comparison, PEDOT‐based devices lack this structural advantage, resulting in higher recombination losses at the anode/active layer interface and a sharper decline in efficiency with decreasing thickness. These differences in vertical morphology highlight the advantages of SAM‐based anodes in enabling efficient charge extraction and greater device stability. By maintaining high performance across a range of active layer thicknesses, SAM‐based devices demonstrate clear potential for advancing QHJ applications. Moreover, the SAM‐based devices exhibit a better thermal stability when compared to PEDOT‐based devices (Supporting Information Figure S18). Consequently, the ability of SAM materials to enhance hole extraction and sustain efficiency under ultra‐thin active layer conditions positions them as promising and durable alternatives to PEDOT for industrial‐scale OSC manufacturing.

## 3 Conclusion

This study demonstrates the significant impact of SAM anodes in improving the performance of QHJ OSCs with ultra‐low donor content. By incorporating only 4 wt% donor material into a NFA matrix, the replacement of the conventional PEDOT anode with a SAM anode enabled a PCE of 7.1%, compared to 4.7% in PEDOT‐based devices. Comprehensive analyses revealed that the SAM anode facilitates vertical phase separation, positioning the donor polymer at the anode interface. This optimized phase distribution enhances charge extraction, suppresses recombination losses, and contributes to significant efficiency improvement. Furthermore, SAM‐based devices can maintain optimal device performance across a wide active layer thickness range (55–180 nm), highlighting their scalability and compatibility with practical fabrication processes. These results underscore the potential of SAM anodes as a key enabler for advancing high‐efficiency organic photovoltaic technologies, especially in devices with minimal donor content, paving the way for scalable and reliable optoelectronic applications.

## 4 Experimental Section

The Supporting Information provides detailed data on the fabrication, characterization, and calculations.

## Supporting Information

Additional supporting information can be found online in the Supporting Information section. **Supporting Fig. S1:** UV‐vis transmittance spectra of PEDOT and SAM films. **Supporting Fig. S2:** EQE spectra and integrated JSC of the devices with SAM‐based anode. **Supporting Fig. S3:** Energy‐level diagram of PM6 [5], PTB7‐Th [5], TFB [6], Poly‐TPD [7], and Y6 [8]. **Supporting Fig. S4:** The J–V characteristics of PM6:Y6 OSCs, utilizing PEDOT as the anode material, were evaluated across various D:A ratios. **Supporting Fig. S5:** The J–V characteristics of PM6:Y6 OSCs, utilizing SAM as the anode material, were evaluated across various D:A ratios. **Supporting Fig. S6:** Variation of photovoltaic parameters in Y6‐based cells with PEDOT and SAM anodes at different D:A ratios. **Supporting Fig. S7:** Transmittance spectra of SAM‐based films at various PM6:Y6 D:A ratios. The AVT of the QHJ film (4:96) is 50.4%, substantially higher than the 35.1% AVT of the BHJ film (40:60). **Supporting Fig. S8:** Variation of VOC × FF in PM6:Y6 cells at different D:A ratios from (a) reference [9] and (b) reference [10]. **Supporting Fig. S9:** UPS spectra of PEDOT‐ and SAM‐based films, (a) Fermi energy was determined by linear extrapolating the high binding energy portion of the spectrum, and (b) HOMO energy level was referred to low binding energy onset. **Supporting Fig. S10:** Contact angle measurements of (a) PEDOT, (b) SAM, and (c) PM6 film with respect to water. Contact angle measurements of (d) PEDOT, (e) SAM, and (f) PM6 films with respect to diiodomethane (DIM). **Supporting Fig. S11:** PL spectra of neat Y6 and PM6 films. **Supporting Fig. S12:** Film‐depth‐dependent light absorption spectra of (a) PEDOT‐ and (b) SAM‐based QHJ samples. **Supporting Fig. S13:** TPC measurements of QHJ cells with PEDOT or SAM anodes. **Supporting Fig. S14:** Light intensity dependence of JSC for PEDOT‐ and SAM‐based devices. **Supporting Fig. S15:** (a) Absorbance of PEDOT‐based QHJ films with thickness ranging from 55 to 95 nm. (b) Absorbance of SAM‐based QHJ films with thickness ranging from 55 to 95 nm. **Supporting Fig. S16:** The J–V characteristics of QHJ cells with PEDOT anode at different active layer thicknesses (55 to 95 nm). **Supporting Fig. S17:** The J–V characteristics of QHJ cells with SAM anode at different active layer thicknesses (55 to 180 nm). **Supporting Fig. S18:** Normalized PCE evolution of PEDOT‐ and QHJ‐based devices under thermal stress (65°C, N_2_ atmosphere). **Supporting Table S1:** Photovoltaic parameters of PEDOT/HJ, SAM/HJ, PEDOT/QHJ, and SAM/QHJ PM6:Y6 OSCs. **Supporting Table S2:** Photovoltaic parameters of SAM‐based QHJ cells blended with various polymer donors (PM6, PTB7‐Th, TFB, or Poly‐TPD). **Supporting Table S3:** Photovoltaic parameters of HJ/QHJ cells reported in the literature, focusing on studies with donor contents below 5%. **Supporting Table S4:** Variation of photovoltaic parameters in Y6‐based cells with PEDOT and SAM anodes at different D:A ratios. **Supporting Table S5:** Contact angle tests were performed using deionized water and DIM droplets on PEDOT and SAM surfaces. Surface energy values calculated using the Wu model (harmonic mean) for various films. Interfacial energy with donor is also provided. The contact angle is average values from 3 measurements. **Supporting Table S6:** Hole mobility, VTFL and Nt of hole‐only SCLC devices with PEDOT and SAM as anodes. **Supporting Table S7:** Variation of photovoltaic parameters for QHJ cells with PEDOT and SAM as anodes under different irradiances. **Supporting Table S8:** The variation in QHJ cells performance with PEDOT and SAM anodes at different active layer thicknesses (55 to 180 nm).

## Funding

NSFC/RGC Collaborative Research Scheme (CRS_CityU104/23), the Research Grant Council of Hong Kong (12300424, 11307323).

## Conflicts of Interest

The authors declare no conflicts of interest.

## Supporting information

Supplementary Material

## Data Availability

The data that support the findings of this study are available in the supplementary material of this article.
