# Supplementary material for: Self‐Assembled Monolayer Anode Enables 7% Efficiency in Y6‐Based Quasi‐Homojunction Solar Cells With 4% Donor Contents
Source: Small Sci. 2026 Mar 19;6(3):e202500552. doi: 10.1002/smsc.202500552 (PMC13097328; doi:10.1002/smsc.202500552)
Supplement: Supplementary file 1 — Supplementary Material [file SMSC-6-e202500552-s001.pdf]

Supporting Information

Self-Assembled Monolayer anode enables 7% efficiency in Y6-based quasi-homojunction solar cells with 4% donor contents

Man Hing Suen[1], Zhuoqiong Zhang[1], Yidan An[2], Pengyu Du[3], Yunfan Wang[2], Yu Tang[1], Chujun Zhang[4], Shibo Wang[5], Tanghao Liu[6], Shanchao Ouyang[2], Guilong Cai[5], Guanghao Lu[3], Hin-Lap Yip[2], Sai Wing Tsang[2], Shu Kong So[1]

[1]Department of Physics and Institute of Advanced Materials, Hong Kong Baptist University, Hong Kong SAR, China.
[2]Department of Materials Science and Engineering, City University of Hong Kong, Hong Kong SAR, China.
[3]School of Chemistry, Xi'an Jiaotong University, Xi'an, China.
[4] Hunan Key Laboratory for Super-microstructure and Ultrafast Process, School of Physics, Central South University, Changsha, China.
[5]Beijing Key Laboratory of Ionic Liquids Clean Process, Institute of Process Engineering, Chinese Academy of Sciences, Beijing, China.
[6]School of Physical Sciences, Great Bay University, Dongguan, Guangdong, China.

Shu Kong So (skso@hkbu.edu.hk) | Zhuoqiong Zhang (18481965@life.hkbu.edu.hk)

Experimental details

Materials: PM6, Y6, and PDINN were purchased from Organtec Ltd. and used as received without further purification to maintain consistency in their chemical properties. Chloroform was obtained from Sigma-Aldrich. PEDOT:PSS (PEDOT) was sourced from Heraeus, while nickel oxide (NiO_x) was purchased from Advanced Electron Technology. Cbz-2Ph was synthesized according to published methods.[1]

Fabrication of Organic Solar Cells (OSCs): The ITO substrate was sequentially cleaned in deionized water, acetone, and ethanol, followed by oxygen plasma for 15 min. For the PEDOT anode, the PEDOT solution was filtered through a 0.45 μm hydrophilic PTFE filter, spin-coated onto the ITO at 4000 rpm for 30 s, followed by annealing at 150 °C for 15 min. The substrates were then transferred to a nitrogen-filled glovebox. The self-assembled material (SAM) anode layer was prepared by two steps. First, NiO_x was dissolved in deionized water (10 mg mL^{-1}), stirred for 1 h, filtered through a 0.45 μm PTFE filter, and spin-coated onto ITO at 1500 rpm for 30 s, followed by annealing at 100 °C for 10 min. Second, Cbz-2Ph, dissolved in isopropyl alcohol and stirred overnight, was spin-coated onto the NiO_x layer at 1500 rpm for 30 s and annealed at 100 °C for 10 min. The active layer was prepared by dissolving PM6 and Y6 in chloroform (18–30 mg mL^{-1}), donor:acceptor (D:A) ratios from 0:100 to 40:60), stirred overnight, and mixed with 0.5% 1-chloronaphthalene for 1 h. This solution was spin-coated onto the hole transport layer (HTL) at 3500 rpm for 60 s and annealed at 100 °C for 10 min. The electron transport layer (ETL) was formed by dissolving PDINN

in methanol (1.5 mg mL^{-1}), stirring for 3 h, and spin-coating onto the active layer at 3500 rpm for 60 s. After a 15 min stabilization period, a 100 nm Ag electrode was deposited via thermal evaporation under high vacuum, using a mask to define an active area of 0.0595 cm^2.

***Characterization of Organic Solar Cells*:** Device performance was assessed by measuring current density–voltage (J–V) characteristics using a Keithley 2400 Source Meter coupled with a Tektronix 2612B Source Meter. A voltage sweep from 0 V to 1.0 V was applied under AM 1.5G illumination, generated by a Newport 96000 150W solar simulator, with light intensity calibrated at each wavelength using a standard single-crystal silicon photovoltaic cell. Measurements were conducted at 25 °C to minimize thermal effects. Dark J–V curves were recorded in a dark box under the same voltage sweep to evaluate intrinsic charge transport properties. UV-vis absorption spectra were obtained using an Agilent Cary 8454 UV-Visible Spectroscopy System. External quantum efficiency (EQE) was measured with an SCS600 Solar Cell Quantum Efficiency Measurement System, employing 150 W xenon and bromine tungsten lamps. Kelvin probe force microscopy (KPFM) data were acquired using a Bruker Multimode 8 atomic force microscope. Ultraviolet photoelectron spectroscopy (UPS) data were collected with a Thermo Fisher Scientific ESCALAB XI+. Photoluminescence (PL) mapping was performed using a WITec alpha300 R Raman System. Transient photocurrent (TPC) measurements were conducted with an ENLITECH PD-RS system. Film depth-dependent light absorption spectroscopy (FLAS) was performed using a

custom setup under oxygen plasma pressure below 30 Pa, as detailed in reference.[2] Film thicknesses were measured with a Veeco Dektak 150 Surface Profiler.

Mobility measurement: Hole-only devices were fabricated using the architectures: ITO/HTL (PEDOT or SAM)/quasi-homojunction (QHJ)/MoO_3/Ag. Mobilities were extracted by fitting the current density-voltage curves using space charge limited current (SCLC). The equation is as follows:

$$J = \frac{9\mu\varepsilon_o\varepsilon_r V^2}{8L^3} \tag{1}$$

$$\frac{I}{A} = \frac{9\mu\varepsilon_o\varepsilon_r V^2}{8L^3} \tag{2}$$

$$I = \frac{9A\mu\varepsilon_o\varepsilon_r}{8L^3} \times V^2 \approx y = a \times x^2 \tag{3}$$

$$\frac{9A\mu\varepsilon_o\varepsilon_r}{8L^3} \approx a \tag{4}$$

$$\frac{8L^3 a}{9A\varepsilon_o\varepsilon_r} \approx \mu \tag{5}$$

where J is current density, V is the voltage, I is the current, μ is the mobility, ε_r is relative dielectric constant of the transport medium (3), and ε_0 is permittivity of free space, A is the area which is 0.0595cm^2, L is the thickness of the active layer.

The trap density (N_t) can be determined using the equation: $V_{TFL} = \frac{eN_t d^2}{2\varepsilon_o\varepsilon_r}$. V_{TFL} is the trap-filled limit voltage, d is the thickness of the active layer, ε_0 is the vacuum permittivity, ε_r is the relative dielectric constant and e is the elementary charge.

Calculation of surface energy and interfacial energy: Wu model (harmonic mean) is used to determine the polar (γ^{p})and dispersive (γ^{d}) components of overall surface energy:

$$\gamma_{water}(1 + \cos\theta_{water}) = \frac{4\gamma_{water}^{d}\gamma^{d}}{\gamma_{water}^{d}+\gamma^{d}} + \frac{4\gamma_{water}^{p}\gamma^{p}}{\gamma_{water}^{p}+\gamma^{p}} \tag{6}$$

$$\gamma_{\mathrm{D}}(1+\cos\theta_{\mathrm{D}})=\frac{4\gamma_{\mathrm{D}}^{\mathrm{d}}\gamma^{\mathrm{d}}}{\gamma_{\mathrm{Dr}}^{\mathrm{d}}+\gamma^{\mathrm{d}}}+\frac{4\gamma_{\mathrm{D}}^{\mathrm{p}}\gamma^{\mathrm{p}}}{\gamma_{\mathrm{D}}^{\mathrm{p}}+\gamma^{\mathrm{p}}} \tag{7}$$

where θ is the contact angle of each thin film; γ is the surface tension of the organic material, which is equal to the sum of the dispersion (γ^{d}) and polarity (γ^{p}) components.

The surface tensions of the liquid droplets for water (γ_{water}) is composed of two components: the polarity ($\gamma_{\mathrm{water}}^{\mathrm{p}}$ and $\gamma_{\mathrm{D}}^{\mathrm{p}}$) and the dispersion ($\gamma_{\mathrm{water}}^{\mathrm{d}}$ and $\gamma_{\mathrm{D}}^{\mathrm{d}}$) components.

$$\gamma=\gamma^{\mathrm{d}}+\gamma^{\mathrm{p}} \tag{8}$$

The interfacial tension between HTL and PM6 was evaluated by the following Equation (9).[3]

$$\gamma_{\mathrm{HTL/PM6}}=\gamma_{\mathrm{HTL}}+\gamma_{\mathrm{PM6}}-\frac{4\gamma_{\mathrm{HTL}}^{\mathrm{d}}\gamma_{\mathrm{PM6}}^{\mathrm{d}}}{\gamma_{\mathrm{HTL}}^{\mathrm{d}}+\gamma_{\mathrm{PM6}}^{\mathrm{d}}}-\frac{4\gamma_{\mathrm{HTL}}^{\mathrm{p}}\gamma_{\mathrm{PM6}}^{\mathrm{p}}}{\gamma_{\mathrm{HTL}}^{\mathrm{p}}+\gamma_{\mathrm{PM6}}^{\mathrm{p}}} \tag{9}$$

where $\gamma_{\mathrm{HTL/PM6}}$ is the interfacial tension between HTL and PM6; γ_{HTL} and γ_{PM6} are the surface tensions of HTL and PM6; the superscript d and p represent the dispersion and polarity components, which are calculated by the above Wu model.

Calculation of average visible transmittance (AVT): The AVT was determined by integrating the spectral transmittance, $T(\lambda)$, weighted by the standard photopic response of the human eye, $V(\lambda)$, and the AM1.5G solar photon flux:[4]

$$AVT=\frac{\int T(\lambda)V(\lambda)\ AM1.5G(\lambda)\ d\lambda}{\int V(\lambda)\ AM1.5G(\lambda)\ d\lambda} \tag{10}$$

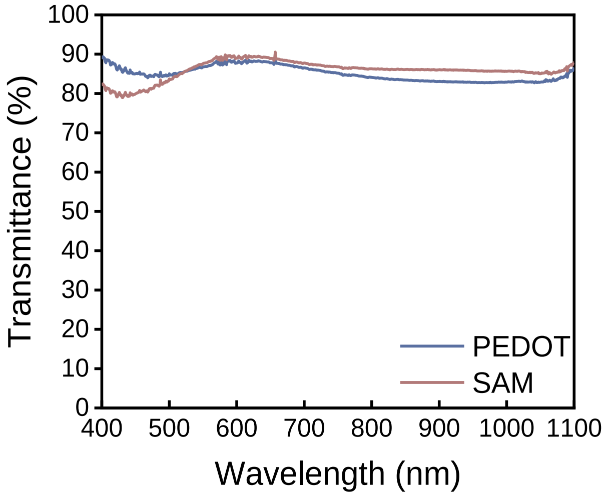

Figure S1. UV-vis transmittance spectra of PEDOT and SAM films.

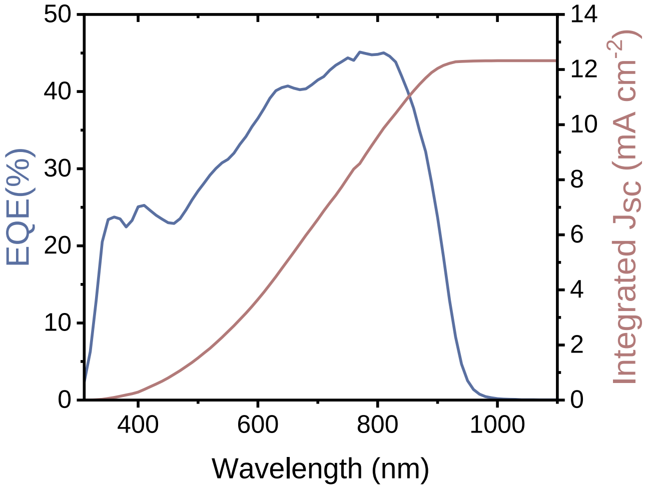

Figure S2. EQE spectra and integrated J_{SC} of the devices with SAM-based anode.

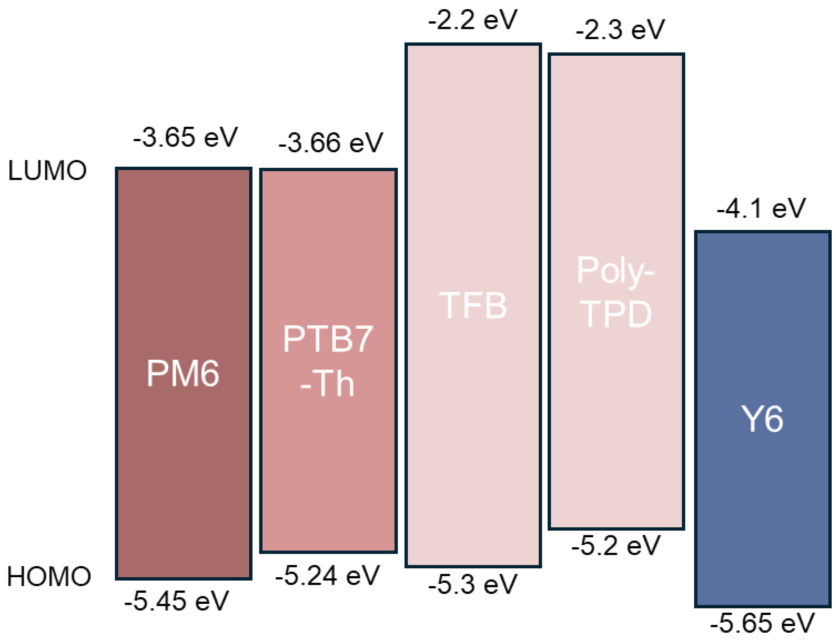

Figure S3. Energy-level diagram of PM6,[5] PTB7-Th,[5] TFB,[6] Poly-TPD,[7] and Y6.[8]

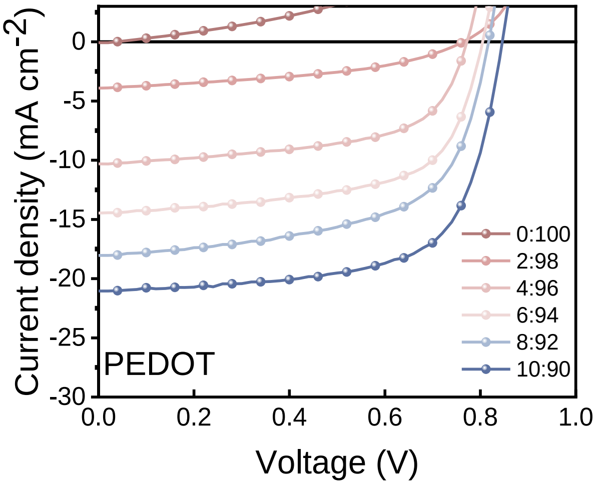

Figure S4. The J–V characteristics of PM6:Y6 OSCs, utilizing PEDOT as the anode material, were evaluated across various D:A ratios.

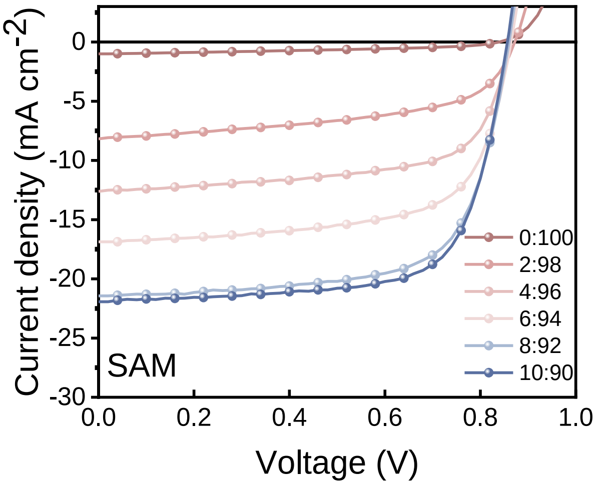

Figure S5. The J–V characteristics of PM6:Y6 OSCs, utilizing SAM as the anode material, were evaluated across various D:A ratios.

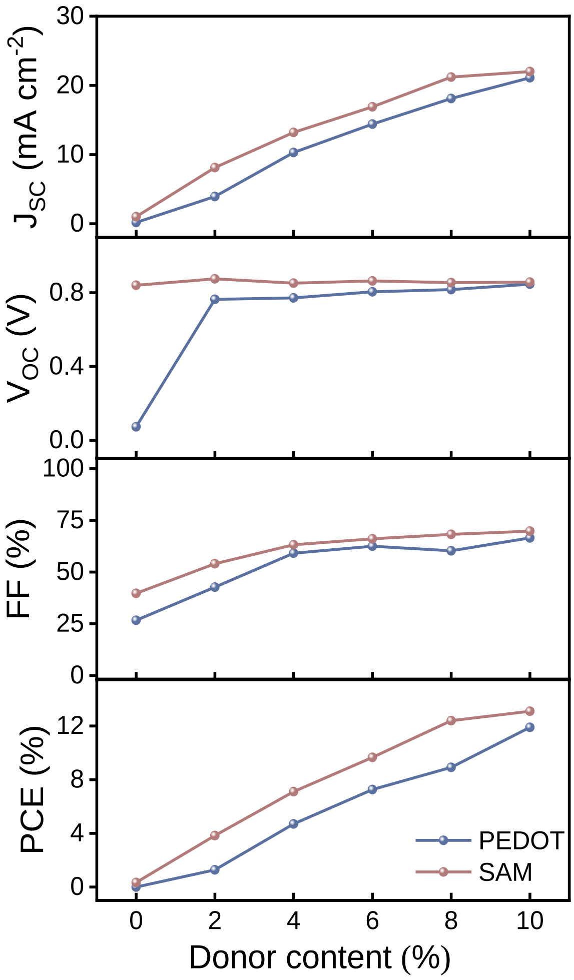

Figure S6. Variation of photovoltaic parameters in Y6-based cells with PEDOT and SAM anodes at different D:A ratios.

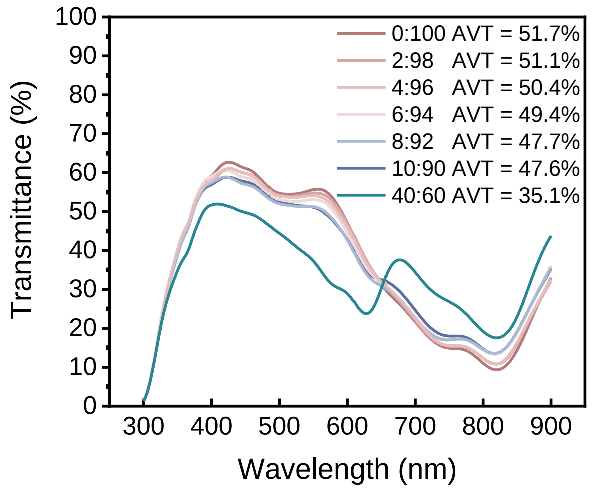

Figure S7. Transmittance spectra of SAM-based films at various PM6:Y6 D:A ratios. The AVT of the QHJ film (4:96) is 50.4%, substantially higher than the 35.1% AVT of the BHJ film (40:60).

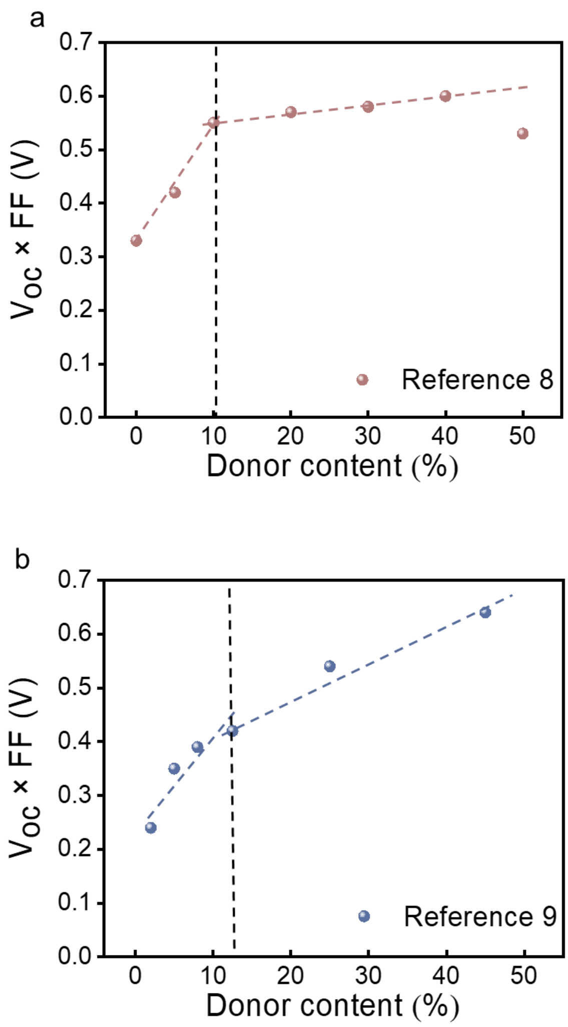

Figure S8. Variation of V_{OC} × FF in PM6:Y6 cells at different D:A ratios from **(a)** reference [9] and **(b)** reference.[10]

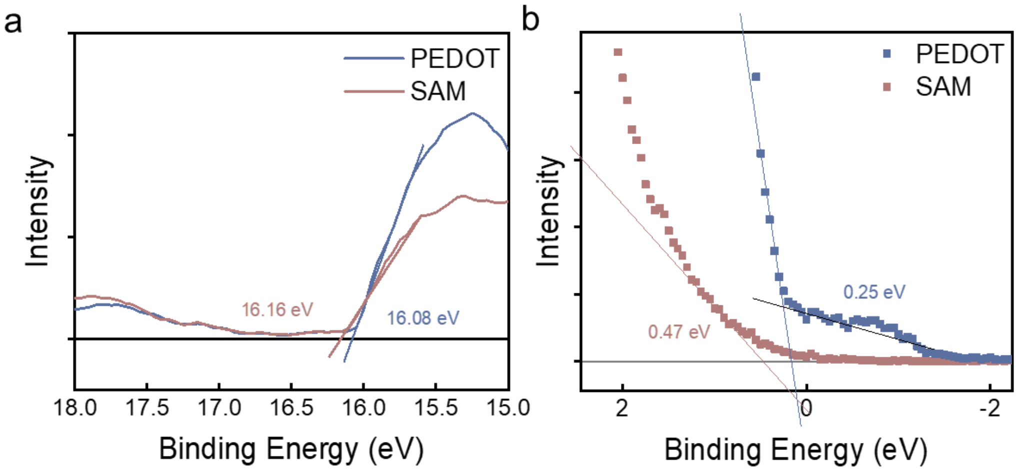

Figure S9. UPS spectra of PEDOT- and SAM-based films, **(a)** Fermi energy was determined by linear extrapolating the high binding energy portion of the spectrum, and **(b)** HOMO energy level was referred to low binding energy onset.

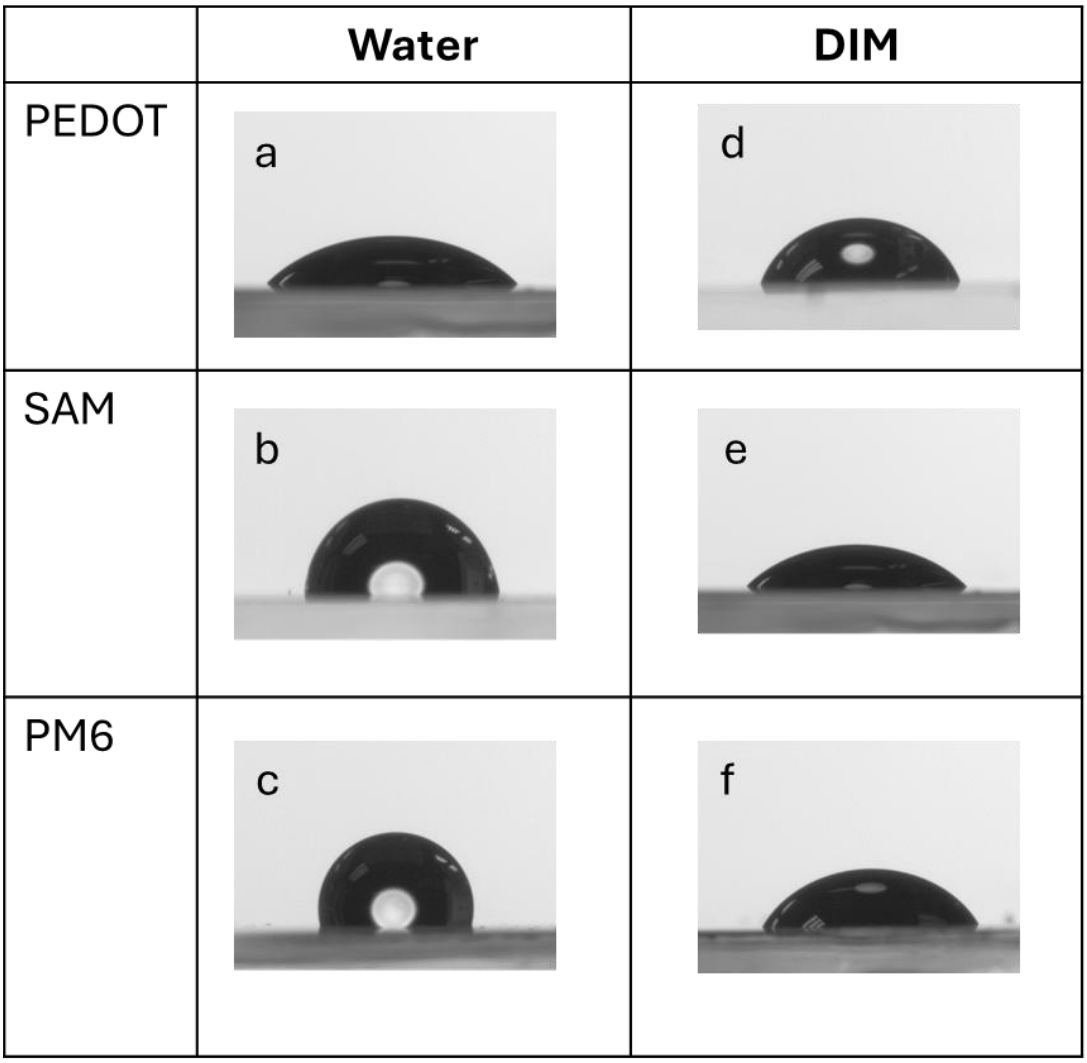

Figure S10. Contact angle measurements of **(a)** PEDOT, **(b)** SAM, and **(c)** PM6 film with respect to water. Contact angle measurements of **(d)** PEDOT, **(e)** SAM, and **(f)** PM6 films with respect to diiodomethane (DIM).

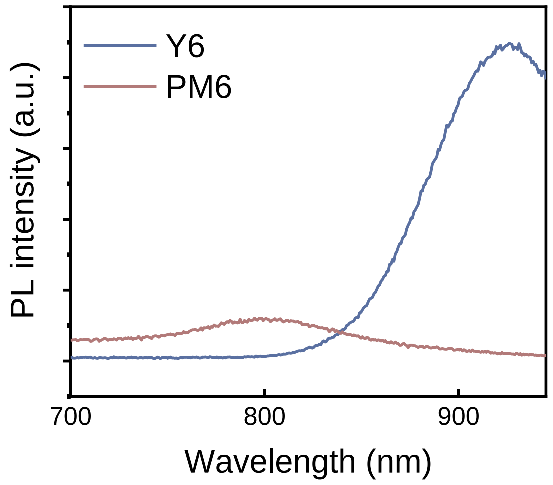

Figure S11. PL spectra of neat Y6 and PM6 films.

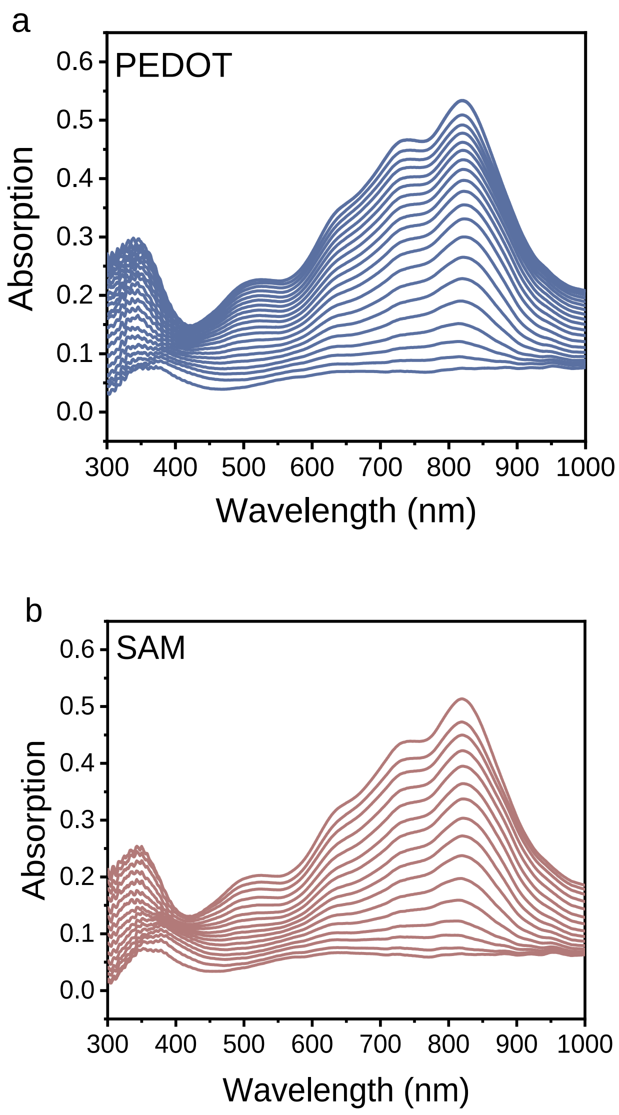

Figure S12. Film-depth-dependent light absorption spectra of **(a)** PEDOT- and **(b)** SAM-based QHJ samples.

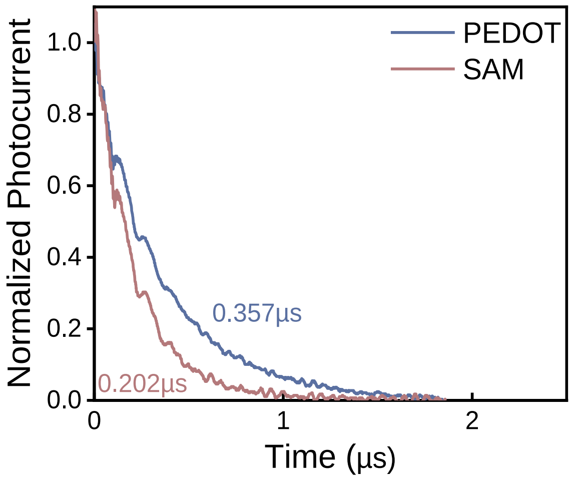

Figure S13. TPC measurements of QHJ cells with PEDOT or SAM anodes.

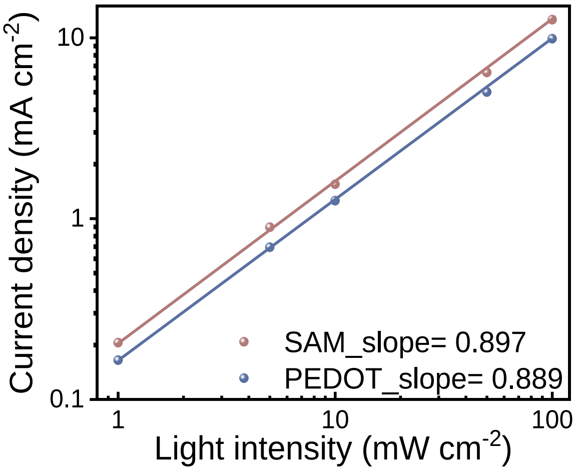

Figure S14. Light intensity dependence of J_{SC} for PEDOT- and SAM-based devices.

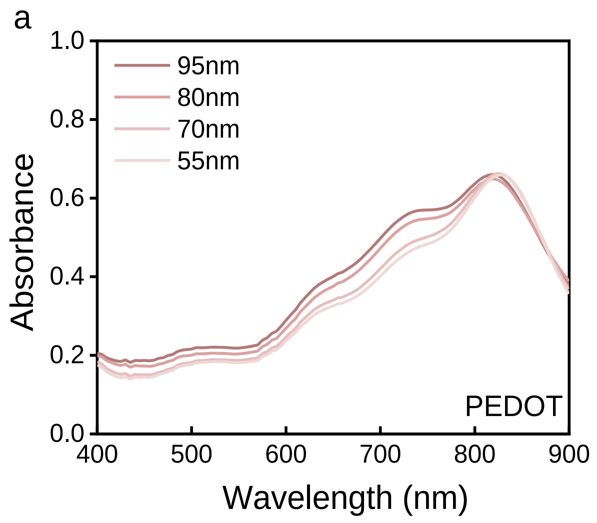

Figure S15. (a) Absorbance of PEDOT-based QHJ films with thickness ranging from 55 to 95 nm.

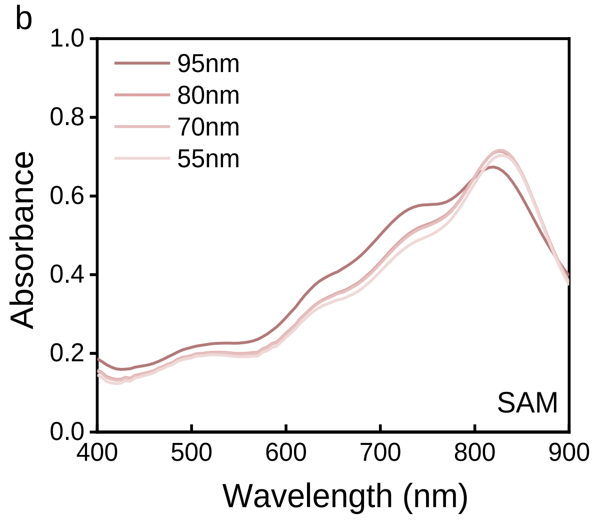

Figure S15. (b) Absorbance of SAM-based QHJ films with thickness ranging from 55 to 95 nm.

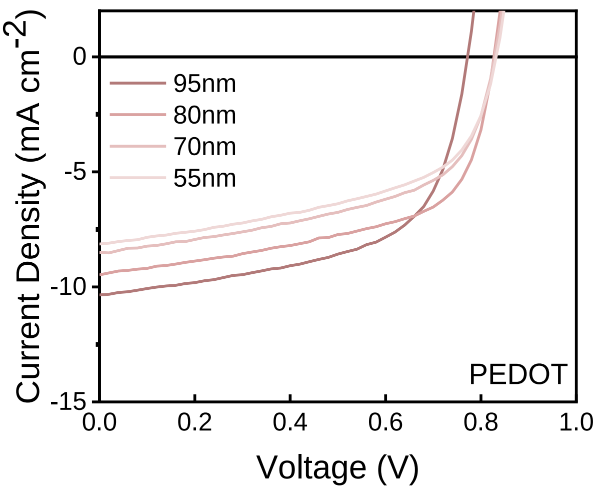

Figure S16. The J–V characteristics of QHJ cells with PEDOT anode at different active layer thicknesses (55 to 95 nm).

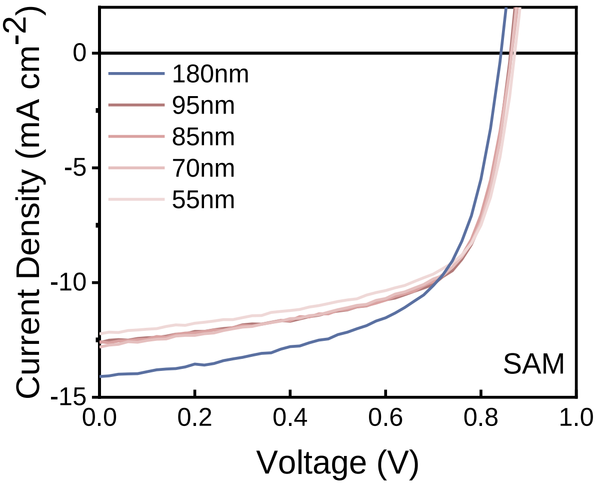

Figure S17. The J–V characteristics of QHJ cells with SAM anode at different active layer thicknesses (55 to 180 nm).

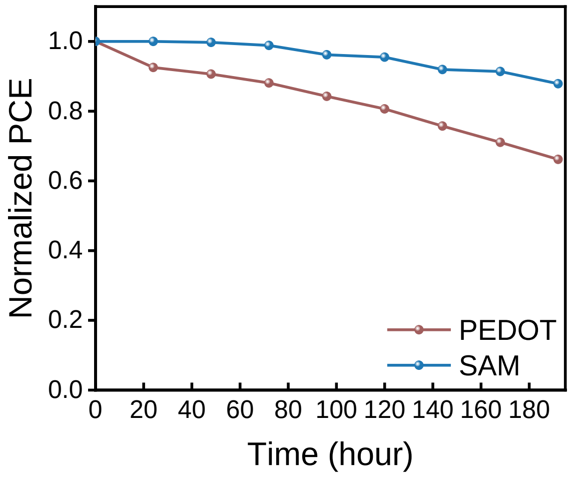

Figure S18. Normalized PCE evolution of PEDOT- and QHJ-based devices under thermal stress (65 °C, N_2 atmosphere).

Table S1. Photovoltaic parameters of PEDOT/HJ, SAM/HJ, PEDOT/QHJ, and SAM/QHJ PM6:Y6 OSCs.

Devices	J_{SC} (mA cm^{-2})	V_{OC} (V)	FF	PCE (%)
PEDOT/HJ	0.2	0.073	0.27	0.003
SAM/HJ	1.0	0.840	0.40	0.3
PEDOT/QHJ	10.3	0.772	0.59	4.7
SAM/QHJ	12.6	0.862	0.65	7.1

Table S2. Photovoltaic parameters of SAM-based QHJ cells blended with various polymer donors (PM6, PTB7-Th, TFB, or Poly-TPD).

Donor material	J_{SC} (mA cm^{-2})	V_{OC} (V)	FF	PCE (%)
PM6	12.6	0.862	0.65	7.1
PTB7-Th	11.8	0.708	0.64	5.3
TFB	3.2	0.797	0.55	1.4
Poly-TPD	3.3	0.634	0.44	0.9

Table S3. Photovoltaic parameters of HJ/QHJ cells reported in the literature, focusing on studies with donor contents below 5%.

References	Anode	Active layer	V_{OC} (V)	FF	PCE (%)	V_{OC} × FF (V)
Our Result	NiO_x/Cbz-2Ph	PM6:Y6 (4:96)	0.862	0.65	7.1	0.56
Small 2025, 2409485[11]	PEDOT:PSS/TFB	Y6	0.75	0.61	2.75	0.46
	MoO_x		0.46	0.48	0.25	0.22
	MoO_x/TFB		0.69	0.50	2	0.35
Adv. Mater. 2022, 34, 2206717[10]	PEDOT:PSS	PTB7-Th:Y6 (1:50)	0.680	0.51	3.45	0.35
		PTB7-Th:Y6 (1:20)	0.700	0.57	7.01	0.40
		PM6:Y6 (1:50)	0.713	0.34	4.18	0.24
		PM6:Y6 (1:20)	0.838	0.42	1.17	0.35
Mater. Horiz., 2023, 10, 1825–1834[12]	CuSCN	Y6	0.79	0.65	4.4	0.51
Energy Environ. Sci 2025,10,1039 [13]	CuSCN	L8BO	0.866	0.61	4.2	0.53
		PM6:L8BO (5:95)	0.863	0.60	7.9	0.52
J. Phys. Chem. Lett. 2021, 12, 5039−5044 [9]	PEDOT:PSS	PM6:Y6 (5:95)	0.81	0.52	4.78	0.42
J. Phys. Chem. C 2022, 126, 20793−20799[14]	PEDOT:PSS	PM6:Y6 (1:99)	0.78	0.47	1.08	0.37
		PM6:Y6 (2:98)	0.77	0.44	1.44	0.34
		PM6:Y6 (5:95)	0.76	0.48	4.83	0.37

Table S4. Variation of photovoltaic parameters in Y6-based cells with PEDOT and SAM anodes at different D:A ratios.

Anode	Donor ratio	J_{SC} (mA cm^{-2})	V_{OC} (V)	FF	PCE (%)
PEDOT	0%	0.2	0.073	0.27	0.003
	2%	3.9	0.764	0.43	1.3
	4%	10.3	0.772	0.59	4.7
	6%	14.4	0.805	0.63	7.3
	8%	18.1	0.817	0.60	8.9
	10%	21.1	0.846	0.67	11.9
SAM	0%	1.0	0.840	0.40	0.3
	2%	8.1	0.875	0.54	3.8
	4%	12.6	0.862	0.65	7.1
	6%	16.9	0.864	0.66	9.7
	8%	21.2	0.855	0.68	12.4
	10%	22.0	0.857	0.70	13.1

Table S5. Contact angle tests were performed using deionized water and DIM droplets on PEDOT and SAM surfaces. Surface energy values calculated using the Wu model (harmonic mean) for various films. Interfacial energy with donor is also provided. The contact angle is average values from 3 measurements.

Solid surface	Water contact angle (Deg)	DIM contact angle (Deg)	Surface energy (mJ m^{-2})	Interfacial energy (mJ m^{-2})
PEDOT	43.5	69.4	54.6	49.4
SAM	91.0	31.1	44.3	3.1
PM6	106.5	59.9	29.8	/

Table S6. Hole mobility, V_{TFL} and N_t of hole-only SCLC devices with PEDOT and SAM as anodes

Anode	Hole mobility (cm^2 s^{-1} V^{-1})	V_{TFL} (V)	N_t (cm^{-3})
PEDOT	8.37×10^{-5}	0.310	1.14×10^{16}
SAM	1.14×10^{-4}	0.248	9.12×10^{15}

Table S7. Variation of photovoltaic parameters for QHJ cells with PEDOT and SAM as anodes under different irradiances

Anode	Light intensity (mW cm^{-2})	J_{SC} (mA cm^{-2})	V_{OC} (V)	FF	PCE (%)
PEDOT	100	9.8	0.788	0.61	4.7
	50	5.0	0.763	0.60	4.6
	10	1.3	0.696	0.57	4.9
	5	0.7	0.675	0.57	5.3
	1	0.2	0.564	0.49	4.6
SAM	100	12.6	0.862	0.65	7.1
	50	6.4	0.851	0.66	7.2
	10	1.5	0.813	0.67	8.4
	5	0.8	0.799	0.66	9.5
	1	0.2	0.751	0.61	9.4

Table S8. The variation in QHJ cells performance with PEDOT and SAM anodes at different active layer thicknesses (55 to 180nm).

Anode	Active layer thickness (nm)	J_{SC} (mA cm^{-2})	V_{OC} (V)	FF	PCE (%)
PEDOT	95	10.3	0.772	0.59	4.7
	85	9.5	0.827	0.58	4.5
	70	8.5	0.828	0.54	3.8
	55	8.1	0.832	0.53	3.6
SAM	180	14.1	0.842	0.60	7.2
	95	12.6	0.862	0.65	7.1
	85	12.6	0.864	0.64	7.0
	70	12.8	0.864	0.63	7.0
	55	12.2	0.871	0.64	6.8

Reference

[1] Y. Wang, W. Jiang, L. Mei, X. Chen, M. Sun, C. Lin, R. Zhang, G. Du, W. Qiu, X. Yang, Q. Fan, H. Yip, F. R. Lin, A. K. -Y. Jen, *Small* **2025**, *21*, 2403233.

[2] Y. Cai, Q. Li, G. Lu, H. S. Ryu, Y. Li, H. Jin, Z. Chen, Z. Tang, G. Lu, X. Hao, H. Y. Woo, C. Zhang, Y. Sun, *Nat. Commun.* **2022**, *13*, 2369.

[3] J. H. Lee, J. Lyu, M. Kim, H. Ahn, S. Lim, H. W. Jang, H. Chung, J. H. Lee, J. Koo, W. H. Lee, *Adv. Funct. Mater.* **2023**, *33*, 2215221.

[4] C. Yang, D. Liu, M. Bates, M. C. Barr, R. R. Lunt, *Joule* **2019**, *3*, 1803.

[5] H. Zhang, H. Yao, J. Hou, J. Zhu, J. Zhang, W. Li, R. Yu, B. Gao, S. Zhang, J. Hou, *Adv. Mater.* **2018**, *30*, 1800613.

[6] Y. J. Han, K. An, K. T. Kang, B.-K. Ju, K. H. Cho, *Sci. Rep.* **2019**, *9*, 10385.

[7] S. Zhang, L. Ye, W. Zhao, D. Liu, H. Yao, J. Hou, *Macromolecules* **2014**, *47*, 4653.

[8] J. Yuan, Y. Zhang, L. Zhou, G. Zhang, H.-L. Yip, T.-K. Lau, X. Lu, C. Zhu, H. Peng, P. A. Johnson, M. Leclerc, Y. Cao, J. Ulanski, Y. Li, Y. Zou, *Joule* **2019**, *3*, 1140.

[9] N. Yao, J. Wang, Z. Chen, Q. Bian, Y. Xia, R. Zhang, J. Zhang, L. Qin, H. Zhu, Y. Zhang, F. Zhang, *J. Phys. Chem. Lett.* **2021**, *12*, 5039.

[10] Y. Wang, M. B. Price, R. S. Bobba, H. Lu, J. Xue, Y. Wang, M. Li, A. Ilina, P. A. Hume, B. Jia, T. Li, Y. Zhang, N. J. L. K. Davis, Z. Tang, W. Ma, Q. Qiao, J. M. Hodgkiss, X. Zhan, *Adv. Mater.* **2022**, *34*, 2206717.

[11] S. McAnally, E. Brooks, O. Lindsay, P. L. Burn, I. R. Gentle, P. E. Shaw, *Small* **2025**, *21*, 2409485.

[12] E. Sağlamkaya, A. Musiienko, M. S. Shadabroo, B. Sun, S. Chandrabose, O. Shargaieva, G. Lo Gerfo M., N. F. Van Hulst, S. Shoaee, *Mater. Horiz.* **2023**, *10*, 1825.

[13] A. Sharma, J. Gorenflot, H. Xu, J. P. Jurado, S. Alam, D. Rosas Villalva, X. Pan, J. Bertrandie, P. D. Nayak, Y. He, M. Alqurashi, Y. Luo, M. R. Andersson, O. J. Sandberg, F. Laquai, D. Baran, *Energy Environ. Sci.* **2025**, *18*, 7610.

[14] B. Zhang, J. C. Bonner, W. Xu, R. T. Piper, L. N. S. Murthy, J. W. P. Hsu, *J. Phys. Chem. C* **2022**, *126*, 20793.
